# Surgeons and scientists: symbiosis in spinal research?

**DOI:** 10.1007/s00586-012-2383-z

**Published:** 2012-06-01

**Authors:** Áron Lazáry, Keita Ito, Stephen Eisenstein, Jeremy Fairbank, Sally Roberts, Dimitris Kletsas, Michelle Kümin, Marco Brayda-Bruno, Péter Pál Varga

**Affiliations:** 1 National Center for Spinal Disorders, Budapest, Hungary; 2 Department of Biomedical Engineering, Eindhoven University of Technology, P.O. Box 513, 5600 MB Eindhoven, The Netherlands; 3 Robert Jones and Agnes Hunt Orthopaedic Hospital, Keele University, Oswestry, UK; 4 Nuffield Orthopaedic Centre, Oxford University Hospitals, Oxford University, Oxford, UK; 5 National Center for Scientific Research “Demokritos”, Athens, Greece; 6 IRCCS Istituto Ortopedico Galeazzi, Milan, Italy; 7 Oxford University, Oxford, UK

## Introduction

Surgical spine research is a growing field involving both surgeons and scientists. There are many surgeons who look to high quality research to improve their quality of care. Although scientists in the field of the spine may also have other scientific motives, most would agree that they are also driven by similar aims. On the one hand, spine surgeons are most intimately involved in the care of these patients and have the broadest surgical and clinical knowledge pertaining to spine ailments. However, most do not have the time, knowledge or training to conduct laboratory-based spine research. The situation is reversed for spine scientists, who in some cases have little contact with clinicians and little understanding of clinical demands. Thus, scientists may provide the innovative key but have less understanding of the problem, while surgeons are constantly faced by the problems, but are frustrated by lack of sufficient “tools”. Both professions require extensive educational training and have extensive time demands. The obvious solution is that they should combine forces to more comprehensively understand spine ailments and to develop surgical/medical methods to better care for patients from the bench to the bedside. This was in fact one of the principles on which this journal was founded [1]. As it was back then, it remains today that spine surgery needs help from other disciplines to arrive at better solutions.

In the modern scientific era, projects are complex involving laboratories, experimental research groups and clinical partners. Although surgeons often have experiences during their training with clinical trials supported by industry, collaboration with laboratory-based scientists requires a different attitude as well as activity. In clinical trials, surgeons often are presented with a finished design with well-determined aims. However, in laboratory-based projects, surgeons must be active partners with specific and relevant comments from the planning to interpretation of the results. Indeed, this type of intense and varied collaboration provides an opportunity to form a reliable and clinically relevant study design, but at the same time it is a difficult challenge for both surgeons and scientists. All partners should be flexible and open to each other’s questions and it is essential that participants learn from each other, with regard to both knowledge and language. These comments are true for clinicians in general, but it is even more so for surgeons because often the interest or the affinity for (basic) science is not encouraged during specialization. This has been well recognized, and calls for research training of surgeons or orthopaedists have already been heard [2, 9]. In addition, such studies may help surgeons to surpass the scepticism on “unfocused” basic research aiming at understanding fundamental mechanisms of tissue structure and function, not immediately translatable into new treatment regimes, as the outcome of these research efforts have very often provided major advancements for the benefit of patients. To help encourage this well-needed collaboration, based on our experiences in numerous international studies, we present some contributing possibilities where spine surgeons can become active partners in scientific research.

## Basic research fields in spine surgery

To make a successful partnership between clinicians and scientists, it is important to realize that there are several varied scientific fields in spine research, each with their own knowledge base and language. Molecular biology studies provide data on special biochemical pathways within the cell. Altered metabolism or other cellular mechanisms can also be demonstrated in different cell/tissue/organ cultures by in vitro assays. In many such studies, these are often sourced from animals. However, as we are interested in the human condition, more and more studies are, quite rightly, being conducted with human tissue samples, mostly collected during spine surgery. Accordingly, surgeons must be encouraged to provide tissue samples, even within the framework of a busy clinical practice.

With the development of biotechnology, population genomic studies have become quite common. As spine surgeons are faced with more and more disorders, which have an apparent significant heritable component, genetic investigations have an increasing role in spine research. There are two major types of genetic studies: genome-wide association studies (GWAS) and candidate polymorphism association studies. In the former, allele variants of genetic markers covering the whole genome are compared between case and control groups. In the latter, candidate gene polymorphisms are selected based on an already demonstrated pathogenic mechanism of the investigated disorder. Genetic studies often require a large population size and well-defined clinical phenotypes. Surgeons are critical to defining the phenotypes.

Another basic research area is biomechanics in which studies are often performed ex vivo and in silico. As the biomechanical role of the spine is important for its function, measurements of (1) physical parameters of vertebrae, (2) soft tissues and (3) spinal muscles can provide significant insight into the clinical condition. The most recently developed computational models are able to simulate the biomechanical conditions of the spine, resulting in the possibility of carrying out not only biomechanical but also mechanobiological studies in silico. Such models are often built on human in vivo data collected in the clinic.

## The role of spine surgeons in different phases of investigation

During preparation of the study design, the spine surgeon should have a significant role to ensure that the main goal of the project is clinically relevant. Discussions with scientists can help to reject ideas, which are beyond technical feasibility. Scientists are usually best at structuring the problem into specific questions and describing possible methods to provide answers. Documentation and structural organization of the collaboration are crucial from the beginning. Although the scientist often works out the details of the study design, clinicians can add practical input, especially in ethical, technical and clinical issues concerning the experimental process preformed in a hospital (e.g. method of patient recruitment, definition of exclusion criteria, surgical techniques and possibilities, ethical approvals, etc.). Indeed, the performance of the methodological steps in theory and practice is often not the same. The appropriate implementation of a study design requires a precise step-by-step protocol created by scientists with respect to the surgeons’ comments. When the protocol is ready, a pilot or a preliminary study could be useful to work out the kinks. Enrollment and informing patients is also a very important role of the surgeon. S/he must incorporate this activity into the research schedule and keep in mind the specific inclusion and exclusion criteria. To get correct samples for effective processing, s/he should be familiar with the laboratory methods and get continuous feedback from the scientists about the samples. Once the data are produced and analysed, the scientific interpretation of results can be performed well by scientists, but surgeons can more clearly “translate” these results for their medical colleagues and patients as well. It is especially imperative when preparing the publication, so that the results of more basic science studies are transferred into clinical practice.

## An example

Most surgeons would insist, if asked, that they do have enquiring minds and are better than mere surgical technicians. However, it is not always straightforward how they can transform this into effective research and here is an anecdotal example from one of the authors of this editorial. As a young orthopaedic surgeon in South Africa, SE observed obvious major differences in the clinical patterns of disease between black and white patients: there was almost no spinal claudication and spinal stenosis in the elderly black patients and sciatica from lumbar disc prolapse was almost unheard of. It was impossible not to be fascinated by the possible genetic, biochemical and physiological origins of these diseases in one group knowing that they were nearly absent in another.

Although there was little contribution to the genetics that he could make, perceiving that most spinal problems in his domain began with some loss of integrity of the intervertebral disc led SE to the notion that he should keep the bits of disc and facet joints that he was removing from discectomy patients and stenosis patients, respectively. He took these to his very patient pathologist. Soon, it became evident that dehydration, chondrocyte loss and loss of staining was characteristic of the excised disc fragments, and the facet joints demonstrated chondrocyte clustering and cartilage loss in what he was to call “chondromalacia facetae”, indistinguishable from osteoarthritis in any synovial joint. In a very busy clinical life, he never got round to publishing anything on the disc, but he did publish on the facet joints [3].

He soon realized that biochemistry of the disc was going to be necessary to explain the histology. It would be necessary to compare pathological with “normal”. He got linked into the kidney and liver transplant programme and achieved consent from the families of young organ donors to allow him to harvest fresh lumbar discs. A colleague biochemist revealed the marked loss of water in the surgical disc specimens, which he provided, compared to the harvested young organ donor discs. They published in 1981 [6]: probably not the only publication of its kind in relation to disc degeneration but, at the time, something of a revelation. How could any surgeon not be fascinated by that? The findings may not lead to some wonderful surgical cure or prevention in the near future, but they may do even better and lead to some non-surgical management in the medium- to long-term future.

By 1985, SE was living and practising in Oswestry, UK, and linked in with a resident scientist there, who became his research partner, a gift of some remarkable circumstances. They have worked together for 26 years on the disc and its degeneration. This has helped improve the understanding of how the disc degenerates [4, 7, 8]. Stimulating an attempt to develop a permanent and effective treatment, there followed a quest for a biological approach [5]. Such work has helped us to realize that a universal silver bullet for discogenic back pain will be elusive and that cell therapy for disc regeneration is extremely challenging in a degenerate disc where the tenuous nutrient supply of the disc is often further compromised. This has resulted in a general refocusing of efforts by many others in spine research towards alternative faster potential mechanical solutions such as injectable gels. The many years of surgeon–scientist collaboration has allowed important conclusions to be achieved and directions for potential solutions to be identified, which would not have been possible without this collaboration.

## Concluding remarks

The increasing significance of laboratory science in clinical research is evident in many journals such as the European Spine Journal. Journals, editors and their referees should celebrate and support these collaborations. Spine societies, both large and small, have a critical role not only as a showcase for such collaborative science, but also as a forum where such collaborations can be encouraged, set up and even funded. Funding agencies should look to these collaborations as the way forward in this area of difficult research. Surgeons and scientists are both faced with this challenge: What better way than to develop a rewarding collaboration with a scientist? It can be an excellent chance to develop organizing, creative and innovative skills that are useful for being not only an investigator, but also a better surgeon. In this way, more significant advances may result from laboratory-based research for the benefit of both and especially for spine patients.
